# Case report: Hemophagocytic lymphohistiocytosis complicated by multiple organ dysfunction syndrome following aseptic encephalitis

**DOI:** 10.3389/fimmu.2023.1296575

**Published:** 2023-12-18

**Authors:** Quan-Ting Chen, Ming-Hua Chen, Yu-Kai Lin, Ren-Hua Yeh, Chun-Chi Lu, Po-Jen Hsiao, Yueh-Feng Sung

**Affiliations:** ^1^ Department of Neurology, Tri-Service General Hospital, National Defense Medical Center, Taipei, Taiwan; ^2^ Department of Internal Medicine, Taoyuan Armed Forces General Hospital, Taoyuan, Taiwan; ^3^ Division of Hematology/Oncology, Department of Medicine, Tri-Service General Hospital, National Defense Medical Center, Taipei, Taiwan; ^4^ Division of Rheumatology/Immunology and Allergy, Department of Internal Medicine, Tri-Service General Hospital, National Defense Medical Center, Taipei, Taiwan

**Keywords:** hemophagocytic lymphohistiocytosis, multiple organ dysfunction syndrome, seizure, status epilepticus, encephalitis

## Abstract

Hemophagocytic lymphohistiocytosis (HLH) is a rare but potentially life-threatening condition caused by excessive immune activation. Secondary HLH is usually triggered by infection, most often from viral infection or malignancy. Here, we present a case of secondary HLH, complicated by multiple organ dysfunction syndrome triggered by critical aseptic encephalitis. A 27-year-old man without any underlying disease presented to our hospital with fever, disturbance of consciousness, and generalized seizures. The patient was diagnosed with aseptic encephalitis with super-refractory status epilepticus. Although antiseizure medications and immunoglobulins were administered, the patient developed multiple organ dysfunction syndrome. HLH was later diagnosed based on hypertriglyceridemia, hyperferritinemia, splenomegaly, cytopenia, and phagocytosis of nucleated cells, as shown by a blood smear of bone marrow aspiration. Treatment with pulse steroid therapy and plasmapheresis was initiated rather than chemotherapy because of the patient’s critical condition. However, the patient died of profound shock and multiple organ failure. Diagnosis of HLH is challenging in patients with severe infections because of similar clinical manifestations and laboratory findings. The early recognition of HLH provides patients with the opportunity to receive appropriate treatment, which can lead to increased survival and remission rates.

## 1 Introduction

Hemophagocytic lymphohistiocytosis (HLH) is a systemic hyperinflammatory syndrome with a high mortality rate. It is a rare disease, with an estimated incidence rate of 1 in 800,000 per year (1). HLH can be classified as either primary (familial) or secondary (acquired). Primary HLH often occurs in early childhood and is caused by pathological immune activation that is often associated with genetic defects in lymphocyte cytotoxicity, whereas secondary HLH is the main cause of adult HLH and is caused by overt immune activation, which can be triggered by infections, or imbalanced immune homeostasis, such as malignancy or rheumatoid disease (2, 3). Both types of HLH may disrupt the normal immune system and result in a vicious cycle of inflammation, eventually leading to a cytokine storm and multiple organ damage (1, 2).

The diagnosis of secondary HLH is challenging because the clinical manifestations and laboratory findings often overlap with severe infections. The HLH-2004 diagnostic criteria were developed for the pediatric populations with HLH (4). According to the diagnostic criteria of HLH-2004, HLH should be genetic mutation associated or should contain at least five of the eight findings, namely, fever, splenomegaly, cytopenia, hypertriglyceridemia and/or hypofibrinogenemia, hemophagocytosis, low or absent natural killer (NK) cell activity, elevated ferritin, and high-soluble CD25 [soluble interleukin-2 receptor (sIL-2R)]. HScore, another scoring system, was developed to provide an effective method to estimate the possibility of secondary HLH in adults (5). The score comprised three clinical (known underlying immunosuppression, temperature, and organomegaly), five biological (cytopenia, ferritin, triglyceride, fibrinogen, and aspartate aminotransferase), and one cytological (hemophagocytic features on bone marrow aspirate) variables and approximately weighted. Currently, there are no standard treatments for adult HLH. However, immunosuppressants may be beneficial, and allogeneic hematopoietic stem cell transplantation (HSCT) may promote disease remission in patients who become reactivated or are refractory to the initial treatment (6). Owing to the high mortality rate, early diagnosis is important for commencing life-saving treatment. Herein, we present a case of secondary HLH complicated by multiple organ dysfunction syndrome (MODS) triggered by critical aseptic encephalitis.

## 2 Case report

A 27-year-old man with no underlying disease presented to our hospital with fever, impaired consciousness, and generalized seizures. One week before admission, the patient had cough and myalgia. He was diagnosed with common cold at a local hospital, where symptomatic treatment was given. On admission, his body temperature was 38.7°C; blood pressure, 137/79 mmHg; heart rate, 97 beats per minute; and respiratory rate, 20 times per minute. Neurological examinations revealed impaired consciousness (Glasgow Coma Scale E3V2M5), intact eyeball movement, normal muscle strength of limbs (Medical Research Council scale grade 5), and bilateral absence of the Babinski sign. His neck was supple. He was intubated with mechanical ventilation later due to deterioration in level of consciousness and status epilepticus.

**Figure 1:**
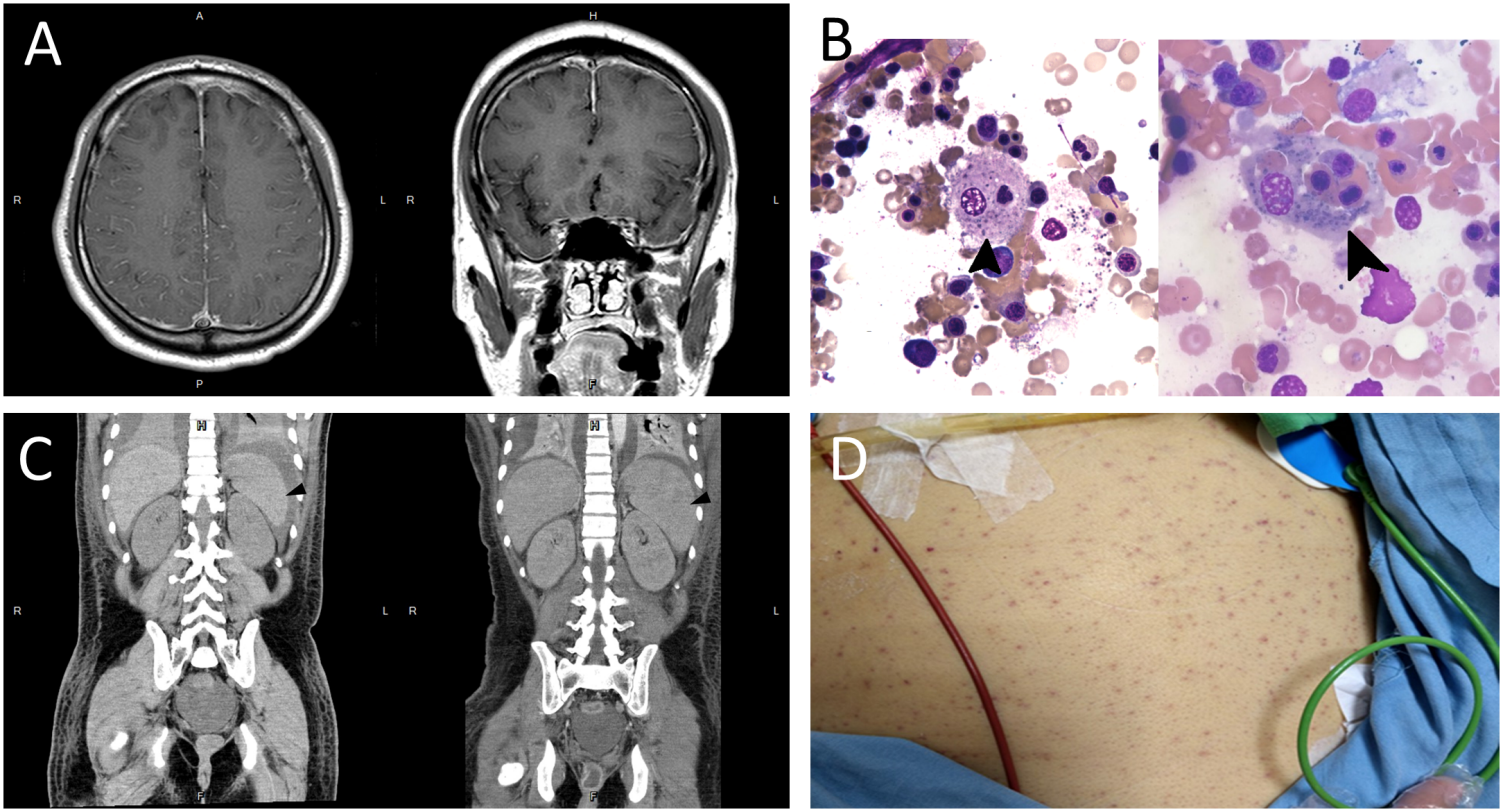
**(A)** T1-weighted brain magnetic resonance imaging with contrast enhancement showing increased vessel enhancement in the cerebral sulci (left panel) and bilateral dura thickening (right panel), suggesting an inflammatory or infectious process. **(B)** Bone marrow smear showing proliferated macrophages with phagocytosis of nucleated cell (arrow, left panel) and platelets (arrow, right panel*)* (Wright-Giemsa stain, × 1000). **(C)** Abdominal computed tomography showing normal spleen size on day 12 of admission (arrowhead, left panel), and splenomegaly (arrowhead, right panel), with the length measuring 12.7 cm on day 18 of admission. **(D)** Diffuse skin petechiae.

Laboratory studies showed leukocytosis (16.6 × 10^3^/µL), elevated aspartate aminotransferase (228 U/L; reference value < 40 U/L), and rhabdomyolysis (creatine kinase [CK], 98540 U/L; reference value, 39–308 U/L). Cerebrospinal fluid (CSF) analysis showed white blood cell count, 144/µL (76% neutrophils, 20% lymphocyte, and 4% histiocytes); red blood cell count, 75/µL; total protein, 100 mg/dL; and glucose, 92 mg/dL (serum glucose, 126 mg/dL). Viral polymerase chain reaction (PCR) assays of CSF were negative for cytomegalovirus, herpes simplex virus (HSV-1 and HSV-2), human herpesvirus 6, varicella zoster virus, enteroviruses, and human parechoviruses. CSF cultures for bacteria, viruses, and fungi, and test for region-specific infections, including dengue virus, Japanese encephalitis virus, tuberculosis, and leptospirosis were all negative. Surveys for autoimmune and limbic encephalitis were negative. The brain magnetic resonance imaging with gadolinium enhancement showed increased vessel enhancement in the cerebral sulci and bilateral dura thickening (**Figure 1A**), suggesting an inflammatory or infectious process. The electroencephalography (EEG) revealed intermittent sharp-and-slow waves and episodic theta slow waves over bilateral hemispheres.

Toxicology screen, blood biochemistry, autoimmune profiles, malignancy surveys, viral hepatitis markers, Epstein–Barr virus (EBV), cytomegalovirus, and human immunodeficiency virus (HIV) screens were unremarkable. Based on the clinical features, CSF data, EEG, and imaging findings, aseptic encephalitis complicated with super-refractory status epilepticus was diagnosed. Despite administering several antiseizure medications, including levetiracetam, topiramate, lacosamide, pregabalin, perampanel, and midazolam infusion, his seizures remained uncontrolled and his consciousness deteriorated. Intravenous immunoglobulin (2 g/kg) was administered on suspicion of seronegative autoimmune encephalitis; however, there was no apparent effect. Bone marrow aspiration was performed because of new-onset bicytopenia (anemia and thrombocytopenia, **Table 1**). The blood smear showed phagocytosis of nucleated cells (**Figure 1B**). Furthermore, new-onset hypertriglyceridemia (478 mg/dL on day 12 of admission; initial value, 84 mg/dL), high serum ferritin level (4501 ug/L; **Table 1**), and new-onset splenomegaly (**Figure 1C**) were found.

**Table 1:** Laboratory results and therapeutic agents used during the course of hospital stay.

	Day 1	Day 2	Day 3	Day 4	Day 5	Day 6	Day 7	Day 8	Day 9	Day 10	Day 11	Day 12	Day 13	Day 14	Day 15	Day 16	Day 17	Day 18	Day 19	Day 20	Day 21
Peak body temperature (°C)	38.2	38.5	< 35.0	< 35.0	37.6	35.2	35.2	39.0	35.4	36.6	37.8	38.5	38.6	39.0	38.5	38.6	37.9	37.6	35.0	35.0	< 35.0
Absolute neutrophil count (10^9^/L)	10.8	4.7	4.3	11.6	11.8			8.3	9.1	11.8	14.0	13.7	8.5	6.5	5.9	4.1	1.9	1.5	1.3	3.2	4.9
Hemoglobin (g/L)	161	133	116	119	110			106	105	104	10.4	99	90	97	96	93	76	79	86	92	99
Platelet count(10^9^/L)	141	128	149	241	218			240	178	123	85	49	81	55	46	89	47	40	52	62	98
Triglyceride (mg/dL)		84										478						473			
Fibrinogen (g/L)												5.19									
Ferritin (μg/L)																4501		3330			
Total bilirubin (mg/dL)			0.3						1.5	3.5	5.4	9.2	10.1	11.4	12.5	12.8	14.4	16.8	14.2	14.8	14.9
Procalcitonin (ng/mL)	0.14			0.37				0.14		0.18		0.37						6.02	8.93	10.76	10.12
Intravenous immunoglobulin				V	V	V	V	V													
Pulse steroid therapy																		V	V	V	
Plasmapheresis																		V	V	V	

Reference normal range:

Body temperature: 35.7°C–37.5°C in ear; absolute neutrophil count: 2.6–6.0 × 10^9^/L; hemoglobin: 132–166 g/L in men; platelet count: 130–400 ×10^9^/L; triglyceride: 40–149 mg/dL; fibrinogen: 2–4 g/L; ferritin: 25–250 μg/L; procalcitonin: < 0.05 ng/mL; total bilirubin: 0.3–1.0 mg/dL.

Finally, HLH was diagnosed based on the diagnostic criteria of HLH-2004 and HScore. Secondary HLH triggered by critical aseptic encephalitis was considered after excluding autoimmune disorders, immunodeficiency, and malignancies. Sepsis and MODS developed, including jaundice (bilirubin, 16.8 mg/dL), acute kidney injury (creatinine, 4.5 mg/dL), acute respiratory distress syndrome (PaO_2_/FiO_2_ ratio < 100), and disseminated intravascular coagulation with multiple skin petechiae (**Figure 1D**). Antibiotics and antifungal agents were administered to control pneumonia, urinary tract infection, leuconostoc bacteremia (detected on day 18 of admission), and *Candida albicans* fungemia (detected on day 20 of admission). After discussion with his family, chemotherapy, such as etoposide, was not used due to the patient’s unstable vital signs and critical condition. Pulse steroid therapy (methylprednisolone, 1 g/day) and plasmapheresis were initiated. However, profound shock without response to vasopressors was noted, and the patient died.

## 3 Discussion

Here, we present a case of HLH complicated by MODS, eventually leading to a fatal outcome. Based on the clinical features, CSF data, EEG, and imaging findings, and exclusion of other autoimmune disorders, immunodeficiency, and malignancies, we believe this was a case of secondary HLH triggered by critical aseptic encephalitis. Although primary HLH is rare in adults (7), the possibility of primary HLH cannot be excluded completely, because genetic test was not performed in this patient.

The prevalence of HLH is extremely low (approximately one per 800,000 people and one to 10 per 1,000,000 children) (1). No standard diagnostic criteria had been established until 1991; the “Histiocyte Society” suggested five diagnostic criteria, namely, fever, splenomegaly, cytopenia, hypertriglyceridemia and/or hypofibrinogenemia, and hemophagocytosis (8). However, its specificity is insufficient to differentiate between HLH and other severe diseases. The revised diagnostic standard was published in 2004 (4) and added three additional criteria: low or absent NK-cell activity, hyperferritinemia, and high sIL-2R levels. According to the HLH-2004 trial, except for verified HLH-associated genetic mutations, the diagnosis of HLH must meet at least five of the eight criteria.

With our patient, six criteria were confirmed, namely, fever, bicytopenia (anemia and thrombocytopenia), hypertriglyceridemia, hemophagocytosis, hyperferritinemia, and splenomegaly. HScore is an effective method to evaluate the possibility of secondary HLH in adults (5, 9). The probability of having hemophagocytic syndrome ranged from <1% with an HScore of ≤90 to >99% with an HScore of ≥250 (5). A validation study of the HScore and the HLH-2004 diagnostic criteria in a multicenter cohort showed that HScore ≥200 demonstrated excellent diagnostic accuracy for HLH (9). The total HScore in our patient was 233 points, indicating that the probability of HLH was approximately 98%–99%.

The patient had an upper respiratory tract infection 1 week before admission, followed by aseptic encephalitis with super-refractory status epilepticus. He was in good health, and no family history and similar symptoms have been noted in the past. Therefore, secondary HLH is more likely than primary HLH in this patient. Features suggestive of a genetic cause include young age at presentation, positive family history, consanguinity, and prominent central nervous system (CNS) disease. Other relevant clinical features such as albinism, inflammatory bowel disease, isolated CNS involvement, and EBV-immunodeficiency may suggest specific genetic causes (7). *PRF1* gene, first described in the pathogenesis and accounting for 20%–40% of hereditary HLH are commonly seen in Asia (10). A large cohort study involving 252 adolescent and adult patients with HLH showed genetic mutations in 18 patients (7.1%), with *PRF1* being most common (11). It is noteworthy that most patients with *PRF1* mutation had viral infection at HLH onset. Other gene mutations linked to familial HLH include *UNC13D*, *STX11*, *STXBP2*, *Rab27a*, *SH2D1A*, or *BIRC4* (12). Mutations in *SH2D1A* are associated with X-linked lymphoproliferative disease type 1, which could result in HLH following EBV infection (13). The findings illustrate that HLH could be the result of a combination of inherited genetic mutations and extrinsic triggers. Therefore, genetic testing in patients with probable HLH should be considered early and it can dramatically affect diagnosis and management.

In a multicenter study of 162 adult patients with reactive hemophagocytic syndrome, a high mortality rate of 20% has been reported in the first month (14). Data suggested an overall mortality rate of 41% in 1109 adults (1). Poor survivals were particularly in those aged over 75 years and those with hematological malignancy (15). In addition to the fatal course of HLH, nonspecific clinical presentation and many mimics can delay diagnosis. The most common mimic is severe sepsis, typically associated with fever, pancytopenia, liver dysfunction, and coagulopathy. Fever with an unknown pathogen and elevated serum ferritin/lactate dehydrogenase levels should raise the suspicion of HLH (16, 17). Macrophage activation syndrome (MAS), a subset of HLH, can present with symptoms similar to those of HLH, including thrombocytopenia, elevated serum levels of aspartate aminotransferase and ferritin, and hypofibrinogenemia (18). The major differences between MAS and HLH are the history of systemic juvenile idiopathic arthritis and adult-onset Still disease in MAS (19) and the relatively better treatment outcomes of MAS (approximately 8% mortality rate) (20). Our patient had no history of rheumatological disorders, and blood tests showed normal autoimmune profiles. Hence, the diagnosis of MAS was unlikely. The early diagnosis of HLH in this patient was challenging because fever accompanied by bicytopenia and splenomegaly might have led us to consider infectious diseases, such as HIV infection (21), and hematological malignancies, such as lymphomas, hairy cell leukemia, and myeloproliferative neoplasms. Furthermore, elevated serum-ferritin levels are commonly observed in patients with infections and sepsis. However, extreme hyperferritinemia should raise a strong suspicion of HLH.

The neurological symptoms of HLH often develop during the course of the disease. However, they can be the initial presenting symptoms (13). Neurological manifestations may include disturbance of consciousness, headache/dizziness, seizure, and psychiatric symptoms (22). We reviewed adult cases suffering from HLH with seizure as the initial presentation of neurological symptoms by searching the keywords “HLH” and “seizure” in the PubMed database (13, 22–28). Only a few cases of primary HLH with seizure as the initial presentation have been reported. The defective genes included *PRF1*, *SH2D1A*, and *MAP2K1* (13, 22, 29). Most patients were diagnosed with secondary HLH with triggers, including infection (EBV was the most common cause), autoimmune disorders, and lymphoma. After excluding patients without detailed descriptions of clinical features, treatment, and outcome, a total of eight patients, including our newly reported patient were summarized in **Table 2**. Mean age of onset was 45 (range: 21 to 63). All patients were diagnosed with secondary HLH, five of them were triggered by infection, two by autoimmune disorders, and one by both autoimmune disorder and infection. The overall mortality rate in adult HLH patients with seizure as the initial presentation was 50%, which was slightly higher than the mortality rate in HLH patients with any clinical presentation (41%) (1).

**Table 2:** Overview of articles reporting on adult patients with hemophagocytic lymphohistiocytosis with initial presentation of seizure/status epilepticus from literature review.

Paper reference	Sex	Onset age (y/o)	Primary/secondary HLH (gene, triggers)	Treatment	Outcome
(23)	Male	38	Secondary (SLE, ankylosing spondylitis)	Steroid, cyclosporine, etoposide	Improved
(23)	Female	21	Secondary (malaria)	Steroid, cladribine, cyclosporine, IVIG	Expired
(23)	Female	62	Secondary (EBV, rheumatoid arthritis)	Steroid, etoposide, rituximab	Expired
(24)	Male	63	Secondary (rheumatoid arthritis)	Steroid, cyclosporine, etanercept, plasmapheresis	Improved
(25)	Male	62	Secondary (SFTS virus)	Steroid, IVIG	Improved
(26)	Female	54	Secondary (tuberculosis)	Anti-TB therapy, steroid, etoposide, cyclosporine	Improved
(27)	Male	30	Secondary (upper respiratory tract infection)	Steroid, IVIG, anakinra, etoposide, IT-MTX	Expired
Current case	Male	27	Secondary (aseptic encephalitis)	Steroid, IVIG, plasmapheresis	Expired

EBV, Epstein–Barr virus; IVIG, intravenous immunoglobulin; IT-MTX, intrathecal methotrexate; SFTS, severe fever with thrombocytopenia syndrome; SLE, systemic lupus erythematosus; TB, tuberculosis.

Early diagnosis of HLH can allow patients to receive appropriate treatment, such as HLH-94 protocol (i.e., etoposide and dexamethasone), with intrathecal therapy for those with CNS involvement before the disease becomes devastating (6, 30). Plasmapheresis or intravenous immunoglobulins may be treatment options for overcoming cytokine storm (30, 31). Additionally, allogeneic HSCT can be considered for patients with refractory or reactivated HLH. The therapeutic benefits of autoimmune antibodies such as rituximab (32), infliximab (33), and Janus kinase 1 and 2 inhibitor ruxolitinib require more clinical evidence (34).

## 4 Conclusion

HLH is a life-threatening disease characterized by uncontrolled immune activation. We present the case of an adult patient with secondary HLH who initially presented with aseptic encephalitis with super-refractory status epilepticus, followed by MODS. The greatest barrier to the early diagnosis of HLH in this patient was the overlapping symptoms of several severe systemic diseases. A high index of suspicion, early diagnosis, and early treatment can reduce mortality and improve outcomes.

## Data availability statement

The original contributions presented in the study are included in the article. Further inquiries can be directed to the corresponding author.

## Ethics statement

The studies involving humans were approved by the Institutional Review Board of Tri-Service General Hospital. The studies were conducted in accordance with the local legislation and institutional requirements. The patient's family provided their written informed consent to participate in this study. Written informed consent was obtained from the individual(s) for the publication of any potentially identifiable images or data included in this article.

## Author contributions

Q-TC: Writing – original draft. M-HC: Writing – review & editing, Writing – original draft. Y-KL: Writing – review & editing, Writing – original draft. R-HY: Writing – original draft, Writing – review & editing. C-CL: Writing – original draft, Writing – review & editing. P-JH: Writing – review & editing. Y-FS: Writing – review & editing.
